# Beyond Efficiency: Considering the Benefits of Residents in the Emergency Department

**DOI:** 10.5811/westjem.38598

**Published:** 2025-05-30

**Authors:** Rachel Elizabeth Armstrong

**Affiliations:** Washington University School of Medicine, Department of Emergency Medicine, St. Louis, Missouri

Determining the productivity and efficiency that emergency medicine (EM) residents add to an emergency department (ED) is an important undertaking. In “Impact of Medical Trainees on Efficiency and Productivity in the Emergency Department: Systematic Review and Narrative Synthesis,” Valentine et al found that residents moderately increase ED productivity while mildly decreasing the efficiency of departments.^1^ As a medical education fellow at Washington University in St. Louis, I have benefitted from the ways institutions value trainees and their educational journey.

The work by Valentine et al underscores the difficulty of balancing educating residents with the reality of ED flow, especially for hospitals considering adding EM rotators or starting a residency. I would add that there are many benefits of having an academic emergency department that go beyond efficiency, including improving patient experience and career satisfaction. For example, Lang et al demonstrated that residents at their institution scored higher on Press Ganey surveys than attendings.^2^ Clinical teaching is linked to ED attendings’ having higher satisfaction with their career, which may prevent burnout.^3^ Let us continue to appreciate the numerous less-tangible ways trainees benefit the department, the hospital system, and patient care that go beyond efficiency and productivity.
